# The Dose and Dose-Rate Effects of Paternal Irradiation on Transgenerational Instability in Mice: A Radiotherapy Connection

**DOI:** 10.1371/journal.pone.0041300

**Published:** 2012-07-24

**Authors:** Safeer K. Mughal, Andrey E. Myazin, Leonid P. Zhavoronkov, Alexander V. Rubanovich, Yuri E. Dubrova

**Affiliations:** 1 Department of Genetics, University of Leicester, Leicester, United Kingdom; 2 Vavilov Institute of General Genetics, Russian Academy of Sciences, Moscow, Russia; 3 Medical Radiological Research Center of the Russian Ministry of Health and Social Development, Obninsk, Russia; Northwestern University Feinberg School of Medicine, United States of America

## Abstract

The non-targeted effects of human exposure to ionising radiation, including transgenerational instability manifesting in the children of irradiated parents, remains poorly understood. Employing a mouse model, we have analysed whether low-dose acute or low-dose-rate chronic paternal γ-irradiation can destabilise the genomes of their first-generation offspring. Using single-molecule PCR, the frequency of mutation at the mouse expanded simple tandem repeat (ESTR) locus *Ms6-hm* was established in DNA samples extracted from sperm of directly exposed BALB/c male mice, as well as from sperm and the brain of their first-generation offspring. For acute γ-irradiation from 10–100 cGy a linear dose-response for ESTR mutation induction was found in the germ line of directly exposed mice, with a doubling dose of 57 cGy. The mutagenicity of acute exposure to 100 cGy was more pronounced than that for chronic low-dose-rate irradiation. The analysis of transgenerational effects of paternal irradiation revealed that ESTR mutation frequencies were equally elevated in the germ line (sperm) and brain of the offspring of fathers exposed to 50 and 100 cGy of acute γ-rays. In contrast, neither paternal acute irradiation at lower doses (10–25 cGy), nor low-dose-rate exposure to 100 cGy affected stability of their offspring. Our data imply that the manifestation of transgenerational instability is triggered by a threshold dose of acute paternal irradiation. The results of our study also suggest that most doses of human exposure to ionising radiation, including radiotherapy regimens, may be unlikely to result in transgenerational instability in the offspring children of irradiated fathers.

## Introduction

The results of recent studies have shown that paternal exposure to ionising radiation and some chemical mutagens not only results in mutation induction in the germ line of directly affected parents, but can also destabilise the genomes of their offspring [1]–[9]. These data challenge the existing paradigm of radiation biology based on the target theory, according to which, mutation induction almost exclusively occurs in directly exposed cells at non-repaired and mis-repaired damaged sites [10]. As far as the offspring of irradiated parents are concerned, the target theory predicts that they should inherit a number of extra mutations from their irradiated parents, therefore implying that the risk of human exposure to ionising radiation is solely attributed to the induction of mutation in the parental germ line [10]. However, new experimental data, showing that in the offspring of irradiated parents mutation rate remains elevated across at least two generations, suggest that the effects of paternal irradiation can manifest over a much longer period of time and hence indicate that the current estimates of genetic risk may require further adjustment.

It should be stressed that despite the results of the abovementioned animal studies, the experimental evidence for transgenerational instability in humans still remains highly controversial. For example, a recent publication showed an elevated frequency of chromosome aberrations among the children of fathers exposed to post-Chernobyl radioactive contamination [11]. On the other hand, using the same technique, Tawn and co-workers failed to detect any significant changes among the children of cancer-radiotherapy survivors [12]. For many reasons, the comparison of human and animal data appears to be problematic, mainly because the doses of paternal exposure analysed in these studies dramatically differed. Thus, the manifestation of transgenerational genomic instability has so far been proven for the offspring of male mice acutely exposed to the doses in excess of 1 Sv [1]–[9], whereas cancer patients receive much smaller scatter doses of fractioned irradiation to distant tissues, and chronic low-dose irradiation represents the main source of human accidental and occupational exposure to ionising radiation [13]. It therefore remains to be established whether low-dose or low-dose-rate parental irradiation can destabilise the genomes of their offspring. Here we have studied the effects of low-dose and low-dose-rate paternal irradiation on the manifestation of transgenerational instability in mice.

## Results

### Experimental Design

**Figure 1:**
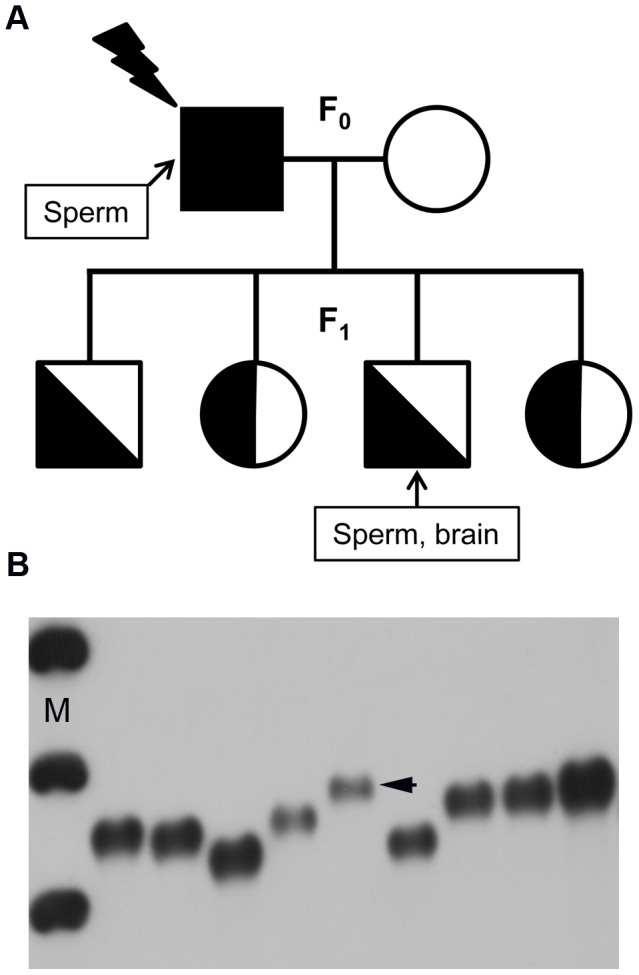
Study design (A) and mutation detection at the *Ms6-hm* ESTR locus by SM-PCR (B). ESTR PCR products from a single heterozygous male are shown together with a 200 bp DNA Step Ladder (M); ESTR mutation is indicated by arrows.

Using Single-Molecule PCR (SM-PCR), mutation frequencies were measured at the *Ms6-hm* ESTR locus in DNA samples extracted from sperm of directly exposed BALB/c male mice, as well as from sperm and brain of their first-generation male offspring (Figure 1A). Similarly to our previous studies on radiation-induced transgenerational instability [1]–[4], [6], [7], irradiated males were mated to control BALB/c females 12 weeks after exposure, thus ensuring that the litters were derived from irradiated A_s_ spermatogonia [14]. For each DNA sample, SM-PCR analysis was conducted on multiple samples, and ESTR mutants were detected on a 40-cm long agarose gel by Southern blot hybridization (Figure 1B). The frequency of ESTR mutation in each tissue was estimated by dividing the number of mutants by the total number of amplifiable DNA molecules.

This study was designed to analyse ESTR mutation induction in the germ line of irradiated males, as well as the transgenerational effects among the offspring of exposed males. Similarly to our previous transgenerational studies [6], [7], the effects of paternal irradiation on the genomes of non-exposed first generation (F_1_) offspring were analysed by establishing the frequency of ESTR mutation in DNA samples extracted from their germ line (sperm) and brain.

### Mutation Induction in the Germ Line of F_0_ Males

**Figure 2:**
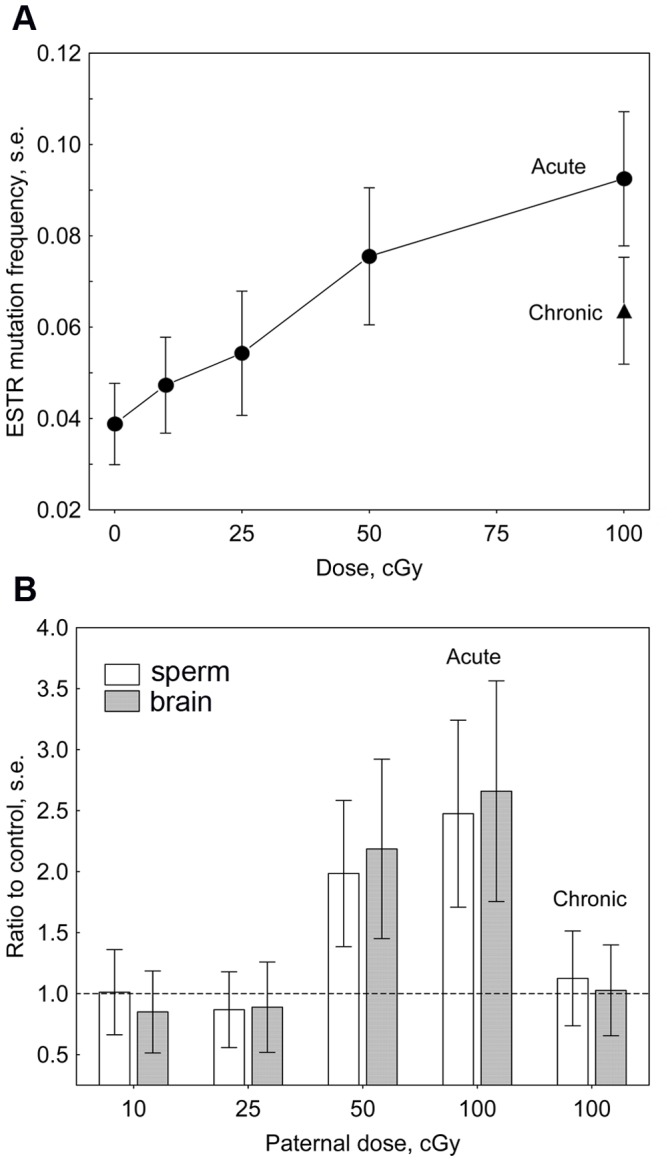
The effects of paternal γ-irradiation on ESTR mutation induction and transgenerational instability. (A) The frequency of ESTR mutation in DNA samples extracted from sperm of control and directly exposed male mice. (B) Transgenerational instability in the first-generation offspring of irradiated male mice. Standard errors are shown.

Table 1 presents a summary of ESTR mutation data. Exposure to 50 and 100 cGy of acute γ-rays resulted in significant increases in ESTR mutation frequency of the germ line of irradiated males. Meanwhile, chronic exposure to 100 cGy was less mutagenic than acute, leading to a marginally significant 1.7-fold increase in the frequency of ESTR mutation. Using the data for acute exposure, we evaluated the dose-response of ESTR mutation induction (Figure 2A). The values of spontaneous mutation frequency, *m_0_* = 0.0388±0.0089 and mean mutation induction, *ind* = (6.85±0.85)×10^−4^ cGy^−1^ were used to estimate the doubling dose for ESTR mutation, *DD* = *m_0_*/*ind* = 57±15 cGy.

**Table 1:** Summary of ESTR mutation data.

Group, tissue^1^	No mutations^2^	Frequency ± s.e.	Ratio to control	*t* 3	*P* 3
Control					
- sperm (4)	20 (516±27)	0.0388±0.0089	–	–	–
- brain (4)	15 (492±27)	0.0305±0.0080	–	–	–
F_0_ sperm					
10 cGy, acute (3)	22 (465±27)	0.0473±0.0105	1.22	0.62	0.5339
25 cGy, acute (3)	17 (313±20)	0.0543±0.0136	1.40	0.95	0.3402
50 cGy, acute (3)	28 (371±23)	0.0755±0.0150	1.95	2.10	0.0359
100 cGy, acute (3)	47 (508±32)	0.0925±0.0147	2.39	3.13	0.0017
100 cGy, chronic (3)	32 (503±27)	0.0636±0.0117	1.64	1.69	0.0920
F_1_					
10 cGy, acute (3)					
- sperm	16 (408±25)	0.0392±0.0101	1.01	0.03	0.9760
- brain	12 (463±27)	0.0259±0.0076	0.85	0.41	0.6803
25 cGy, acute (3)					
- sperm	14 (416±25)	0.0337±0.0092	0.87	0.40	0.6905
- brain	10 (369±23)	0.0271±0.0087	0.89	0.28	0.7756
50 cGy, acute (3)					
- sperm	29 (377±23)	0.0769±0.0151	1.98	2.18	0.0294
- brain	25 (375±23)	0.0667±0.0139	2.19	2.25	0.0248
100 cGy, acute (3)					
- sperm	26 (271±18)	0.0959±0.0199	2.48	2.62	0.0088
- brain	24 (296±20)	0.0811±0.0174	2.66	2.64	0.0085
100 cGy, chronic (3)					
- sperm	16 (367±23)	0.0436±0.0112	1.12	0.34	0.7359
- brain	17 (543±30)	0.0313±0.0078	1.03	0.07	0.9417

1 Number of animals is given in brackets.

2 Number of amplifiable DNA molecules (± s.e.) is given in brackets.

3 Student’s test and probability for difference from controls.

### ESTR Mutation Frequencies in the Offspring of Irradiated Males

To analyse the transgenerational effects of paternal irradiation, ESTR mutation frequencies were evaluated in DNA samples extracted from sperm and brain of their F_1_ offspring and compared with those in controls (Table 1, Figure 2B). The frequency of ESTR mutation in both tissues was significantly elevated in the offspring of male mice that received 50 and 100 cGy of acute γ-rays. The magnitude of transgenerational increases in the two groups did not significantly differ (*t* = 0.76, *P* = 0.45 and *t* = 0.65, *P* = 0.52 for sperm and brain, respectively). In contrast, acute exposure to smaller doses did not destabilise the F_1_ genomes; importantly, the same was also true for the F_1_ offspring of male mice that received a chronic exposure 100 cGy of low dose-rate γ-rays.

## Discussion

This study was specifically designed to analyse the dose- and dose-rate effects of irradiation on mutation induction in the germ line of exposed male mice and the manifestation of transgenerational instability in their first-generation offspring. Our results offer further insights onto the yet unknown mechanism underlying the phenomenon of radiation-induced transgenerational instability in mammals and also provide a plausible explanation for the existing controversy regarding the experimental evidence for these effects in humans.

The analysis of ESTR mutation induction in the germ line of acutely exposed male mice has revealed a linear dose-response within the dose range of 10 to 100 cGy. These data are in line with the results of our previous studies on radiation-induced ESTR mutation [15], [16]. Thus, the value of the doubling dose for ESTR mutation is close to that for the BALB/c strain obtained using a pedigree-based approach (57±15 cGy and 88±29 cGy, respectively; *t* = 0.95; *P* = 0.32; the pedigree data taken from ref. 16). These data, together with the results of our previous studies on the genetic effects of exposure to chemical mutagens [17], [18] therefore show that the SM-PCR technique provides a valuable approach for monitoring ESTR mutation induction in the mouse germ line. Here we also report that chronic exposure to low-dose-rate γ-rays is less efficient than acute. This result is in line with the Russell data showing that chronic γ-irradiation produces fewer mutations in the male mouse germ line than are induced by an acute dose rate [19], [20]. However, we did not confirm the results of our previous pedigree-based study showing similar increases in ESTR mutation rate in the germ line of CBA/H male mice following acute and chronic exposure to X- and γ-rays [21]. Future work should therefore address this issue in detail.

The main result of this study shows that paternal exposure to low-/medium-doses of acute γ-rays, as well as chronic γ-irradiation does not destabilise the F_1_ genomes. Although acute exposure to doses of 10–25 cGy is considered to be mutagenic [10] and, according to our data, results in a steady increases in ESTR mutation frequency in the germ line of irradiated males (Figure 2A), its transgenerational effects appear to be negligible. The same is also true for the effects of low-dose-rate chronic irradiation; with an effect being observed for the directly irradiated males but not in the male F_1_ offspring. Meanwhile, the magnitude of transgenerational instability observed in germ line and brain genomes of the offspring of male mice exposed to 50 and 100 cGy of acute γ-rays appears to be roughly similar (Figure 2B). Taken together, these data imply that, in sharp contrast to the direct effects of ionising radiation where the yield of induced mutations is proportional to the dose- and dose-rate of exposure, the manifestation of transgenerational instability is triggered by a threshold dose of acute paternal irradiation. According to our results, the threshold dose for transgenerational effects following paternal exposure to acute low-Linear Energy Transfer (LET) γ-irradiation should not exceed 50 cGy. It should be noted that two recent studies have also found no evidence for the long-term effects of whole-body acute γ-irradiation on the manifestation of radiation-induced chromosomal instability in the bone marrow of mice exposed to doses below 100 cGy [22], [23].

The data on threshold-like events resulting in the long-term destabilization of the descendants of irradiated cells/organism provide important clues regarding the mechanisms underlying this phenomenon. They raise a possibility that transgenerational instability manifested in the offspring could be triggered by a stress-like response induced in the germ line of parents acutely exposed to high-dose irradiation. Given the results of our previous studies showing that paternal high-dose exposure to an alkylating agent ethylnitrosourea or some mutagenic anticancer drugs can also destabilise the F_1_ genomes [8], [9], it would appear that the triggering of an instability signal is not attributed to a specific subset of DNA lesions, but instead depends on the amount of generalized DNA damage. As far as the exposure to ionising radiation is concerned, the total amount of radiation-induced DNA damage increases practically linearly within a very wide range of doses [24]. If correct, then the threshold-like events described here most probably reflect the inability of exposed cells to cope with a certain amount of DNA lesions induced over a short period of time following acute irradiation. This notion is fully supported by our current data, showing a complete lack of transgenerational instability among the offspring of male mice exposed to very low dose-rate chronic irradiation, whereas the same dose of 100 cGy of acute paternal exposure substantially destabilises the F_1_ genomes. The two experiments only differ by the duration of paternal exposure, when the dose of 100 cGy was delivered over the period of time of 2 min and 2 weeks for acute and chronic irradiations, respectively. It would therefore appear that the amount of DNA damage inflicted over a certain period of time plays an important role in triggering the F_1_ genomic instability. The irradiated cell may be effectively ‘stunned’ by a certain amount of DNA damage delivered over a short period of acute exposure, which exceeds DNA-repair capacity. The very survival of such a cell would require a substantial overstretch of all systems involved in DNA repair and some other pathways of cellular homeostasis, which, in turn, may necessitate a profound changes in the pattern of gene expression. Indeed, microarray analysis has revealed that high- and low-dose irradiations affect the expression of practically non-overlapping sets of genes in mammalian cells [25]. Some of the changes occurring after acute exposure may become permanent and affect the epigenetic landscape of the surviving cell and its descendants. If this is the case for paternal irradiation, then the abovementioned epigenetic changes occurring in the germ line of exposed males may be passed to the offspring and somehow destabilise their genomes.

The results of our study provide a plausible explanation for the existing controversy surrounding the data on transgenerational effects in humans. Given that in all current radiotherapy regimens normal tissues are shielded and seldom receive doses in excess of 10 cGy per single procedure [13], it would appear highly unlikely that the exposure to such small doses can result in transgenerational effects among their children. However, as the total absorbed dose at the end of a fractionated treatment to the shielded germ line can reach 100 cGy [13], it may appear that such paternal exposure could destabilise the F_1_ genomes in humans. Although the transgenerational effects of such fractioned irradiation, delivered on the daily basis have not been analysed here, the results of numerous studies show that the mutagenicity of fractioned low-LET irradiation is similar to that for low dose-rate chronic and much lower than following acute exposure [19], [20], [26], [27]. It therefore appears that our data on transgenerational instability among the offspring of chronically-irradiated males may also reflect the effects fractionated exposure. Future studies should address the effects of low-dose fractionated irradiation on the manifestation of transgenerational instability.

In summary, the results of our mouse study, showing no effects of low-/medium-dose acute and low-dose-rate paternal irradiation on the manifestation of transgenerational instability are important in furthering our understanding of the delayed genetic effects in humans. Judging from our data, it would appear that one of the most common types of relatively high-dose paternal exposure in humans, radiotherapy [13] may not destabilise the F_1_ genomes. Given the results of our recent study showing that the offspring of female mice exposed to 1 Gy of acute X-rays are genetically stable [7], this may also be true for maternal irradiation. However as the transgenerational effects of maternal exposure to smaller doses of ionising radiation remain unknown, this issue requires further analysis. It should also be stressed that as our data describe the effects of low-LET paternal irradiations (X-/γ-rays), they do not address the issue of transgenerational instability following exposure to high-LET sources, such as α-particles and fission neutrons, the efficiency of which remains to be established.

## Materials and Methods

### Ethics Statement

All animal procedures were approved by the Ethical Committee of Vavilov Institute of General Genetics.

### Animals

Male 6-week-old BALB/c inbred mice (Vavilov Institute of General Genetics colony) were used in this study. They were housed at the Vavilov Institute of General Genetics animal facility. Eight-week-old male mice were γ-irradiated at the Panorama Facility, Obninsk, Russia using a ^137^Cs source at absorbed dose-rates of 4.26 cGy min^−1^ (10 cGy acute), 50 cGy min^−1^ (25–100 cGy acute), and 0.005 cGy min^−1^ (100 cGy chronic). The absorbed dose was assessed using 27012 and DKS-101 dosimeters. Caudal epididimy were collected from the irradiated F_0_ males 12 weeks after exposure; caudal epididimy and brain were also taken from the 8 week old male F_1_ offspring of exposed and non-exposed control mice. Control samples were taken from 4 non-exposed males.

### DNA Preparation

Sperm DNA and brain samples were prepared in a laminar flow hood as previously described [4], [6]. Approximately 5 µg of each DNA sample was digested with 20 units of *Mse*I for 2 h at 37°C; *Mse*I cleaves outside the ESTR array and distal to the PCR primer sites used for PCR amplification and was used to render genomic DNA fully soluble prior to dilution. Each digested DNA sample was diluted to ∼10 ng ml^−1^ prior to mutation analysis.

### Mutation Detection

The frequency of ESTR mutation at the *Ms6-hm* ESTR locus located on the mouse chromosome 4 was evaluated using SM-PCR [4]–[6], [7], [9]. The *Ms6-hm* ESTR locus was amplified in 10 µl reactions using 0.4 µM flanking primers HM1.1F (5′-AGA GTT TCT AGT TGC TGT GA-3′) and HM1.1R (5′-GAG AGT CAG TTC TAA GGC AT-3′). The Expand High-fidelity PCR System (Roche, Mannheim, Germany) was used (0.035 U µL^−1^) with 1 M betaine and 200 µM of each deoxynucleotide triphosphate. Amplification was carried out at 96°C (20 s), 58°C (30 s), and 68°C (3 min) for 29 cycles on a PTC-225 DNA Engine Tetrad (MJ Research, Waltham, Mass).

For each DNA sample, multiple aliquots containing on average one Ms6-hm molecule were amplified. PCR products were then resolved on a short agarose gel and detected by Southern blot hybridisation. For each 96-well reaction plate the number of amplifiable DNA molecules was estimated by Poisson analysis. To increase the robustness of the estimates of individual ESTR mutation frequencies, on average, 133 amplifiable molecules were analysed for each tissue in each male mouse. Positive PCR products were subsequently analysed for the presence of ESTR mutations on a 40-cm-long agarose gel and detected by Southern blot hybridisation as previously described [15]. The frequency of ESTR mutation, *μ* in each tissue was estimated by dividing the number of mutants, *m* by the total number of amplifiable DNA molecules, *λ*. The standard error of mutation frequency, *seμ* was estimated as:
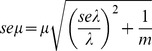
where *seλ* is the standard error of the number of amplifiable DNA molecules estimated using a modified approach proposed by Chakraborty [28].
